# 非小细胞肺癌PD-L1免疫组织化学检测规范中国专家共识

**DOI:** 10.3779/j.issn.1009-3419.2020.101.43

**Published:** 2020-09-20

**Authors:** 中国抗癌协会肿瘤病理专业委员会肺癌学组, 中国抗癌协会肺癌专业委员会, PD-L1检测共识专家组

## 1 引言

近年来，以程序性死亡受体1(programmed death-1, PD-1)/PD配体1(PD ligand 1, PD-L1)免疫检查点抑制剂为主的免疫治疗在晚期肺癌中取得了突破性的进展，改变了该领域的治疗格局，为患者带来了更多生存获益。虽然对于免疫治疗适宜人群筛选和疗效预测的生物标志物越来越多，但PD-L1仍是目前应用最为广泛的指标。免疫组织化学(immunohistochemistry，IHC，简称免疫组化)检测是评估肿瘤组织PD-L1表达状态的一种有效且最常用方法，广泛应用于包括非小细胞肺癌(non-small cell lung cancer, NSCLC)等在内的多种恶性肿瘤中，以识别或辅助预测可能从免疫治疗中获益的患者。

为更好地指导临床检测，中国抗癌协会肿瘤病理专业委员会肺癌学组召集国内部分相关病理专家及临床专家，就PD-L1免疫组化检测的适应证、检测评估复杂性、多样性及临床检测应用注意事项等，参考国内外相关指南及目前中国临床使用和研究现状，讨论并制定本共识供国内同行参考，以期进一步规范和指导肺癌免疫治疗中PD-L1免疫组化检测的临床实践和应用。

## 2 国内肺癌免疫检查点抑制剂适应证

随着PD-1/PD-L1免疫检查点抑制剂临床试验及应用的广泛开展，大量相关药物批准上市，分别获得美国食品药品监督管理局(Food and Drug Administration, FDA)、欧盟(European Communities, CE)认证和国家药品监督管理局(National Medical Products Administration, NMPA)的批准。按照《中国非小细胞肺癌免疫检查点抑制剂治疗专家共识(2019版)》 ^[1]^内容推荐，目前针对晚期NSCLC驱动基因阴性患者，中国已有多个PD-1/PD-L1抑制剂适用于一线、二线或以上治疗^[2]^，其中PD-L1检测结果可以作为伴随诊断指导晚期NSCLC患者一线接受帕博利珠单抗单药或联合治疗。PD -L1检测结果也可作为补充诊断为晚期NSCLC患者接受纳武利尤单抗作为二线或以上治疗提供信息。

## 3 免疫检查点抑制剂预测标记物PD-L1指标使用

随着PD-1/PD-L1免疫检查点抑制剂获批，NSCLC患者PD-L1免疫组化检测试剂等也随适应证需要作为伴随诊断或补充诊断而相应获批^[3, 4]^。其中最大特点是各个药物分别对应不同的PD-L1试剂克隆或平台，且其判读阈值也各不相同^[5]^。表 1汇总了FDA、CE认证和NMPA对NSCLC患者免疫治疗时所需使用PD-L1检测试剂和检测平台的获批情况(截止2020年5月31日)。本共识建议选择我国NMPA批准的免疫组化检测试剂盒或抗体试剂，具体推荐内容详见表 1。

**1:** 不同国家地区NSCLC患者PD-L1检测试剂和检测平台获批情况 Approval status of PD-L1 assays and platforms in selected countries/region

试剂名称	抗体克隆号	检测平台	药物通用名	FDA及获批阈值	CE认证及获批阈值	NMPA及获批阈值	推荐级别
PD-L1 IHC 22C3 pharmDx	22C3 鼠单克隆一抗	Dako Autostainer Link48	帕博利珠单抗	伴随诊断 TPS≥1%	认证 TPS≥1%、 TPS≥50%	批准TPS≥1%	优先 推荐
22C3抗体试剂	22C3 鼠单克隆一抗	Dako Autostainer Link48	帕博利珠单抗	/	/	批准TPS≥1%	优先推荐(需LDT确认)
PD-L1 IHC 28-8 pharmDx	28-8 兔单克隆一抗	Dako Autostainer Link48	纳武利尤单抗	补充诊断(非鳞状NSCLC) TC≥1%、TC≥5%、TC≥10% 伴随诊断(NSCLC) TC≥1%(纳武利尤单抗联合伊匹单抗)	认证(非鳞状NSCLC) TC≥1%、TC≥5%、TC≥10%	批准 (非鳞状NSCLC) TC≥1%	推荐
VENTANA PD-L1 (SP142) assay	SP142 兔单克隆一抗	Ventana BenchMark ULTRA	阿替利珠单抗	补充诊断/伴随诊断 TC≥50%或IC≥10%	认证 TC≥50%或IC≥10% TC≥1%或IC≥1%	/	可考虑
VENTANA PD-L1 (SP263) assay	SP263 兔单克隆一抗	Ventana BenchMark ULTRA	纳武利尤单抗	/	认证 TC≥1%、≥5%、 ≥10%	/	可考虑
			帕博利珠单抗		认证 TC≥50%——一线治疗 TC≥1%——二线治疗		
			度伐利尤单抗		认证，TC≥1%		
“/”代表未获批；TPS：肿瘤比例评分；TC：肿瘤细胞；IC：免疫细胞；LDT：实验室自建检测；FDA：美国食品药品监督管理局；CE：欧盟；NMPA：国家药品监督管理局。PD-L1: programmed cell death-ligand 1. NSCLC: non-small cell lung cancer.							

除PD-L1(22C3)试剂盒获批以外，其浓缩液也于2020年5月获NMPA批准作为体外诊断试剂，用于实验室自建检测(laboratory developed tests, LDT)。根据各自实验室性能确认结果，PD-L1浓缩抗体经相应比例预稀释后，于相应的免疫组化染色机(Dako Autostainer Link48)染色，其判读结果可作为PD-L1伴随诊断，以指导临床评估是否可使用帕博利珠单抗治疗NSCLC患者，如果判读结果TPS≥1%，则认为该样本存在PD-L1阳性表达结果。

PD-L1免疫组化检测的获批试剂对应药物各有不同^[1, 4, 6-8]^，因此各检测平台和试剂间的相关性和一致性也是临床医生与病理医生关注的重点。PD-L1检测抗体在NSCLC中相关性和一致性的研究至今已有很多，其中比较有影响力的是由国际肺癌研究协会(International Association for Study of Lung Cancer, IASLC)牵头进行的蓝印计划(The Blueprint Project)I和II^[9, 10]^。研究对比了克隆号28-8、22C3、SP263、SP142及73-10(蓝印计划研究II期)这4种-5种检测抗体，结果显示：①28-8、22C3、SP263对肿瘤细胞染色的阳性百分比相似，一致性较高；②SP142对肿瘤细胞染色的灵敏度较低，与其他抗体间的一致性低；③73-10相比于其他抗体表现出更强的敏感性。④病理学家对PD-L1在肿瘤细胞中表达分数评估一致性较高，而对免疫细胞PD-L1表达结果评估一致性较差。国内也开展了一些关于PD-L1检测抗体相关性和一致性的研究^[11]^，结果与国际报道一致，显示22C3、28-8和SP263对肿瘤细胞染色一致性较高(*ρ*=0.729-0.809)，SP142的肿瘤细胞着色较弱，灵敏度较低。不过此类研究目前仅局限于分析验证领域，尚缺乏不同检测抗体间一致性的临床疗效验证。

## 4 PD-L1检测适用人群及检测时机

### 4.1 PD-L1检测适用人群

PD-L1检测的目的是提供PD-1/PD-L1抑制剂治疗的疗效预测性信息，因此，PD-L1检测的患者选择应以PD-1/PD-L1抑制剂的获批适应证为主要依据。至目前为止，获NMPA批准用于治疗NSCLC的PD-1/PD-L1抑制剂有帕博利珠单抗、纳武利尤单抗及度伐利尤单抗^[12-15]^。综合考虑药物及检测试剂在国内外的获批情况，虽然临床用药前对PD-L1检测的适用人群有一定规定，但是在病理临床实践中，有时不能判断或不能及时知晓其用药选择。因此，建议在兼顾检测成本、临床送检需求与实际操作的前提下，尽量为所有可能具有免疫治疗机会的NSCLC患者提供PD-L1免疫组化检测结果。

### 4.2 PD-L1检测时机

PD-L1的检测结果可以指导一线用药，因此推荐在晚期NSCLC患者初诊时进行PD-L1免疫组化检测。《2019版中国非小细胞肺癌免疫检查点抑制剂治疗专家共识》^[1]^和《2019版中国临床肿瘤学会(CSCO)原发性肺癌诊疗指南》^[16]^提出，基于KEYNOTE-024^[17, 18]^和KEYNOTE-042^[19]^研究的结果，帕博利珠单抗单药作为一线治疗时，需要检测患者的PD-L1表达，且与美国国立综合癌症网络(National Comprehensive Cancer Network, NCCN)NSCLC临床实践指南^[20]^推荐内容一致，将PD-L1检测与表皮生长因子受体(epidermal growth factor receptor, *EGFR*)、间变性淋巴瘤激酶(anaplastic lymphoma kinase, *ALK*)等基因检测列入同等地位。当PD-1/PD-L1抑制剂与含铂双药联合用于一线治疗或用于后线治疗时，PD-L1表达的检测并非强制，但该检测可能会提供有用的信息^[16]^。因此，晚期NSCLC患者确诊后进行PD-L1免疫组化检测和驱动基因检测同等重要。

## 5 PD-L1免疫组化检测

### 5.1 PD-L1检测标本要求

检测标本选择是有效评估肿瘤组织PD-L1表达的关键步骤。影响该领域临床实践的问题集中于标本类型与判读标准等方面。

#### 5.1.1 标本类型

基于多项临床研究^[21, 22]^的结果，利用组织学标本(来自手术切除和活检)检测的PD-L1表达水平对PD-1/PD-L1免疫检查点抑制剂治疗的疗效预测作用已经得到了国内外监管机构的认可，在临床中优选组织学标本PD-L1检测结果为临床用药提供参考。考虑到标本可及性，仍然有许多患者只能提供细胞学标本检测PD-L1表达^[5]^。多项研究^[10, 23-25]^显示，细胞蜡块和对应的组织学标本检测肿瘤细胞PD-L1表达结果具有较高的一致性，支持使用细胞学包埋蜡块检测肿瘤细胞PD-L1表达。免疫细胞计数相对复杂，需要考虑免疫细胞的真实有效性^[10]^，因此暂不推荐细胞学包埋蜡块用于免疫细胞PD-L1表达判读。至于细胞学涂片样本，尽管有限的研究报道组织学标本与细胞涂片肿瘤细胞PD-L1表达结果一致性也较高^[26, 27]^，但由于缺乏充足的研究数据支持及目前尚无获批的用于细胞涂片的试剂产品，因此目前临床实践暂不推荐使用细胞涂片检测肿瘤PD-L1表达。

#### 5.1.2 标本检测前处理

与病理行业对所有免疫组化检测指标的质控要求一致，PD-L1检测前处理原则应遵循病理规范化诊断总则要求，具体包括取材前剖开固定、冷缺血时间(最好保证冷缺血时间不超过30 min)、标本固定方式(10%中性缓冲福尔马林固定液)、固定时间(活检标本6 h-24 h，手术标本12 h-48 h，最长不超过72 h)、蜡块储藏时间、白片储藏时间等。PD-L1检测标本的保存时间会影响IHC染色效果^[28]^。有研究^[29]^对比≤3年和 > 3年所获得的肿瘤标本PD-L1表达情况，结果显示，≤3年内获得的标本与近期获取的标本之间PD-L1表达率一致性较高(76.2%)，而存储时间 > 3年的标本中PD-L1高表达比例明显下降。因此，建议优先选择近期标本检测，尽量避免使用 > 3年的标本应用于PD-L1检测^[30]^。另外，为保证染色质量与检测结果的准确性，推荐使用新鲜切取的白片(切片1周内)进行免疫组化检测^[31]^，临床实践中应尽量避免长时间存储白片。目前尚无脱钙标本对免疫组化检测PD-L1结果影响的充足研究数据，但参考分子检测质控要求并结合试剂使用说明，推荐尽量避免使用脱钙标本进行常规PD-L1指标检测；若使用脱钙标本检测，建议在报告中予以体现或备注说明。

### 5.2 PD-L1检测的判读标准

由于免疫组化结果判读主要通过人为及半定量判断，具有一定主观性，因此，PD-L1检测应在有资质的实验室由经过PD-L1判读培训的病理医师进行诊断^[32]^。根据判读标准，除了SP142需要至少50个肿瘤细胞外，其他抗体需要不少于100个肿瘤细胞。使用不同克隆的试剂盒检测NSCLC肿瘤组织的PD-L1表达水平时，判读标准存在细微差异，详见下述判读注意事项。总体上讲PD-L1检测都需要病理医生在光学显微镜下评估肿瘤区域染色情况，评估对象及染色部位包括单独评估肿瘤细胞膜或同时评估肿瘤细胞膜和免疫细胞膜和/或细胞质，计算染色比例，并根据染色比例为临床治疗提供参考信息^[30]^。各检测试剂盒名称、平台、检测细胞类型、判读阈值等信息请参见表 1。使用各PD-L1克隆号抗体检测NSCLC判读注意事项具体如下。

#### 5.2.1 22C3抗体

使用该抗体检测时只观察肿瘤细胞膜染色情况，细胞质阳性着色忽略不计。部分或全部细胞膜表达任何线性或颗粒状染色的肿瘤细胞均算作阳性细胞，且PD-L1(22C3)是唯一一个以TPS(任何强度的部分或完全膜染色的肿瘤细胞占标本中所有肿瘤细胞的百分比)作为表达结果的抗体。TPS < 1%诊断为阴性表达，TPS≥1%诊断为阳性表达，其中TPS 1%-49%为低表达，TPS≥50%为高表达，上述数值区间对临床用药有指导意义。实际诊断中也可以给出具体数值如5%、8%或30%、75%等。TPS≥1%是NMPA批准药物对应的PD-L1表达界值。

#### 5.2.2 28-8抗体

该抗体判读标准与22C3相同，只是关注的表达数值区间不同。药物临床试验涉及到的相关界值有肿瘤细胞(tumor cell, TC)PD-L1表达≥1%、≥5%、≥10%，同样实际诊断中可以类似22C3一样给出具体数值。TC≥1%是NMPA批准药物对应的PD-L1表达界值。

#### 5.2.3 SP263抗体

结果判读与22C3、28-8标准大致相同，唯一不同之处在于若SP263仅有肿瘤细胞基底膜染色不能算作阳性，需同时侧膜染色时才可判为阳性。药物研发相关界值有TC≥1%、≥5%、≥10%、≥50%，建议检测时给出TC具体数值即可。

#### 5.2.4 SP142抗体

使用SP142抗体检测时，除了TC结果判读标准与22C3、28-8相同外，还需检测免疫细胞(immune cell, IC)染色情况，这也是目前使用该抗体检测NSCLC的特殊之处。IC计数范围包括肿瘤内和/或紧邻的肿瘤周围连续间质中，除外坏死区域；IC群体包括淋巴细胞、巨噬细胞、树突状细胞和粒细胞；染色模式为胞膜或/和细胞质内深棕色点状或线状，可以是单细胞或/和聚集体形式；IC评分方法为任何强度PD-L1染色的免疫细胞所占区域/肿瘤区域，再乘以百分数。具体评分时TC、IC各采用4等级评分，其中TC膜染色4等级：0%- < 1%为TC0、≥1%- < 5%为TC1、≥5%- < 50%为TC2、≥50%为TC3；IC染色4等级：0%- < 1%为IC0、≥1%- < 5%为IC1、≥5%- < 10%为IC2、≥10%为IC3。评分采用逐级评分步骤，首先评估TC染色，若TC≥50%，则不必再评估IC。若TC < 50%，需进一步评估IC染色。药物研发相关界值为TC≥50%或IC≥10%以及TC≥1%或IC≥1%。鉴于目前尚无中国获批的应用于NSCLC的对应药物，建议检测时给出TC和IC各自评分和/或级别即可。

### 5.3 检测的规范化报告模式推荐

PD-L1 IHC检测的结果报告中除了常规病理报告中的基本信息外，还应包括标本信息、PD-L1检测流程相关信息以及PD-L1检测结果三部分。标本信息包括采集部位、标本类型、蜡块存储时间以及脱钙情况等；PD-L1检测流程相关信息包括PD-L1检测所用平台、PD-L1检测所用抗体克隆号以及检测标本质控等；PD-L1检测结果包括PD-L1表达相关参数及结果。报告模板示意见图 1。

**1:**
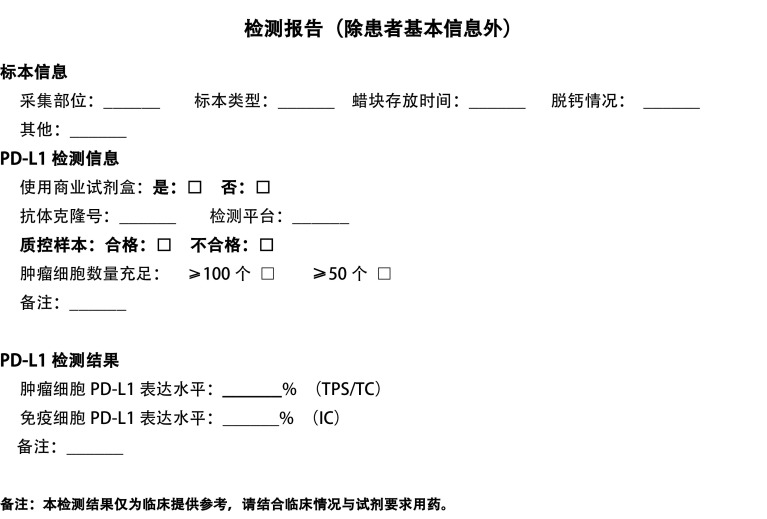
PD-L1检测报告模板示意 PD-L1 test report template

### 5.4 LDT

LDT是指由实验室内部研发、确认和使用，以诊断为目的的体外诊断方法。对于不具备使用获批商业试剂盒检测条件或检测瘤种情况下，实验室经方法学确认合格的LDT或可作为PD-L1检测的备选方案。严格来讲，开展LDT实验室需经过相关医学实验室质量和能力认可(如ISO15189认可或CAP认证等)，完成必要的抗体性能确认和免疫组织化学实验室检查能力考核合格后，人员方能授权上岗。现阶段中国大陆地区开展PD-L1免疫组化LDT检测尚处于摸索阶段，需不断积累相关临床实践数据及经验方可给出共识推荐内容^[33, 34]^。

### 5.5 检测质控

无论选择何种PD-L1免疫组化检测试剂及平台，都需要行之有效的质量控制体系。其中，实验室内部质控是确保PD-L1免疫组化检测质量的关键，也是实验室外部质评的前提。

#### 5.5.1 实验室内部质控

室内质控的主要目的是确保检验步骤的准确性和实验室每次检测结果的准确性和可重复性。首先，必须确保实验室的PD-L1检测试剂盒/相关试剂可靠，并符合NMPA及相关法规要求，且实验室需要提供技术检测和评判(如同步对照[On-slide]染色等)合格的证据。其次，除保证检测前样本处理达到要求外，室内质控常规应设立阳性及阴性对照，对照建议以采用：①已知的PD-L1强表达(≥50%)、中-弱表达(1%-49%)以及阴性表达(< 1%)肿瘤标本(可考虑TMA芯片形式)；②具有可重复性和检查上下限的正常组织，如扁桃体(网状隐窝上皮弥漫细胞膜强染色，而表层鳞状上皮细胞阴性；生发中心淋巴细胞或巨噬细胞中-弱表达，滤泡间细胞大多数阴性)；③正常胎盘组织(滋养层细胞强表达，绒毛间质细胞不表达)。另外还应进行不同检测方法的比对、不同检测人员的比对、新试剂盒/试剂性能的验证以及定期抽检等。最后，还需要进行规范必要的实验室记录以便溯源，定期进行人员培训、考核和再培训，数据总结和分析、仪器设备维护等。

#### 5.5.2 实验室外部质评

参与室间质评活动是实验室外部质评的主要方式。实验室应定期参加PD-L1检测室间质评活动，每年至少2次。室间质评可通过参加国内权威机构举办的室间质评活动来完成，也可通过与其他实验室(如已获得资格认可的实验室、使用相同检测方法的实验室等)比对的方式确定检测结果的可信度。

## 6 PD-L1表达异质性相关科学问题

尽管PD-L1表达对PD-1/PD-L1免疫检查点抑制剂的疗效预测作用已经获得国内外监管机构的一致认可，但在研究及临床应用中仍有一些与PD-L1表达相关的科学问题需要进一步探讨。

### 6.1 PD-L1表达的瘤内异质性

目前，临床获取组织学标本的方式主要有手术切除标本和活检标本(包括肿物穿刺)。了解标本间PD-L1表达的异质性对临床检测有着重要的指导作用。不同研究中，PD-L1表达在手术标本与活检标本间存在一定异质性^[35-38]^，国内相关研究同样显示类似结果^[39, 40]^。一项研究(*n*=239)发现单个活检标本与手术标本间的不一致率较高；为降低风险，推荐至少取4个活检标本进行检测^[41]^。另一项研究(*n*=268)发现PD-L1表达值最高的活检标本与手术标本的一致性最高，且活检标本数量不足将影响PD-L1表达检测的准确性^[42, 43]^。因此，使用活检样本检测PD-L1时，应注意尽量获取足量的活检标本(建议4个活检标本)，建议对所取组织全部进行PD-L1检测，对不同组织表达情况，应重视表达率最高的组织块(条)的检测数值，或许更加接近肿瘤本身PD-L1的表达状况。当然相关临床实践问题需待进一步研究不断充实并完善。

### 6.2 PD-L1表达的瘤间异质性

不同研究发现PD-L1表达在原发灶与淋巴结转移灶/局部复发灶/远处转移灶之间存在差异，不一致率为11.4%-39% ^[29, 44-50]^；但似乎肿瘤自然进展转归的病灶(未经治疗复发或转移灶)与原发灶之间的差异并不显著^[44, 51]^。而多项研究^[51-61]^均发现，患者在接受抗肿瘤治疗(化疗、放化疗、靶向治疗、免疫治疗等)前后，PD-L1表达水平会出现不同程度的变化，但PD-L1表达高低差异程度目前无明确定论。

PD-1表达是动态变化的，但由于现有研究样本量有限，临床试验入组标准不统一，目前各个研究的结论存在一定不一致之处，对此仍有待进一步探索研究。因此，目前建议临床实践中尽可能使用免疫治疗前最近期的标本进行PD-L1检测，若近期标本不可及，可考虑采用3年内的组织/细胞蜡块标本。

## 7 总结

PD-L1检测作为预测PD-1/PD-L1免疫检查点抑制剂用于晚期NSCLC一线、二线及以上治疗的疗效预测生物标志物，已在绝大多数病理实验室中得以应用，并被临床医生广泛应用于筛选或辅助判断免疫治疗中可能获益的患者。规范化操作程序和标准化结果判读能提高PD-L1检测的准确性和可重复性。另外，加强临床与病理的沟通交流将有助于获取更准确的PD-L1检测结果及对治疗疗效的客观评价。本共识后续将根据临床诊治进展及临床实践不断完善，进一步更新。

**Table:** 非小细胞肺癌PD-L1免疫组织化学检测规范中国专家共识专家组成员(按姓氏汉语拼音排名)

	医院	姓名
参与专家	南京大学医学院附属鼓楼医院	樊祥山
参与专家	复旦大学附属中山医院	侯英勇
参与专家	四川大学华西医院	蒋莉莉
参与专家	复旦大学附属肿瘤医院	李媛
参与专家	北京大学肿瘤医院	林冬梅
参与专家	中山大学附属肿瘤医院	林素暇
参与专家	上海交通大学附属胸科医院 上海市肺部肿瘤临床医学中心	陆舜
参与专家	中国科学院大学附属肿瘤医院	孙文勇
参与专家	华中科技大学同济医学院附属同济医院	王国平
参与专家	吉林大学第一医院	王银萍
参与专家	北京医院	王征
参与专家	同济大学附属上海市肺科医院	武春燕
参与专家	山西省肿瘤医院	郗彦凤
参与专家	广东省人民医院 广东省医学科学院	颜黎栩
参与专家	中国医科大学附属盛京医院	杨向红
参与专家	湖北省肿瘤医院	岳君秋
参与专家	广东省人民医院 广东省肺癌研究所	张绪超
参与专家	天津医科大学附属肿瘤医院	赵纲
